# Supplementary material for: The C-terminal tail of the bacterial translocation ATPase SecA modulates its activity
Source: eLife. 2019 Jun 27;8:e48385. doi: 10.7554/eLife.48385 (PMC6620043; doi:10.7554/eLife.48385)
Supplement: Supplementary file 2. [file elife-48385-supp2.docx]

**Data collection and refinement statistics for the crystal structure of SecAΔMBD.**

|  | **SecAΔMBD** |
| --- | --- |
| **Wavelength** | 0.9795 |
| **Resolution range** | 47.5 - 3.5 (3.625 - 3.5)* |
| **Space group** | C 1 2 1 |
| **Unit cell** | 163.1 106.8 76.0 90 102.8 90 |
| **Total reflections** | 32115 (3197)* |
| **Unique reflections** | 16106 (1609)* |
| **Multiplicity** | 2.0 (2.0)* |
| **Completeness (%)** | 99.66 (99.81)* |
| **Mean I/sigma(I)** | 13.51 (1.51)* |
| **Wilson B-factor** | 132.20 |
| **R-merge** | 0.03651 (0.5532)* |
| **R-meas** | 0.05163 (0.7823)* |
| **R-pim** | 0.03651 (0.5532)* |
| **CC1/2** | 1 (0.672)* |
| **CC*** | 1 (0.897)* |
| **Reflections used in refinement** | 16106 (1609)* |
| **Reflections used for R-free** | 785 (89)* |
| **R-work** | 0.2316 (0.3407)* |
| **R-free** | 0.2927 (0.3891)* |
| **CC(work)** | 0.900 (0.645)* |
| **CC(free)** | 0.869 (0.405)* |
| **No. non-hydrogen atoms** | 6182 |
| **Protein residues** | 777 |
| **RMS(bonds/angles)** | 0.011/1.47 |
| **Ramachandran (%)** favoured/allowed/outliers | 91.42/5.07/3.51 |
| **Rotamer outliers (%)** | 3.64 |
| **Clashscore** | 14.32 |
| **Average B-factor** | 158.55 |
| **PDB** | 6GOX |

*Statistics for the highest-resolution shell are shown in parentheses.
