# Supplementary material for: The C-terminal tail of the bacterial translocation ATPase SecA modulates its activity
Source: eLife. 2019 Jun 27;8:e48385. doi: 10.7554/eLife.48385 (PMC6620043; doi:10.7554/eLife.48385)
Supplement: Supplementary file 3. [file elife-48385-supp3.docx]

**Table describing SAXS data collection and processing details for SecA, SecAΔMBD and SecAΔCTT.**

| **Sample Details** |  |
| --- | --- |
| **SecA** |  |
| Organism | *Escherichia coli* |
| Source | *Escherichia coli* |
| UniProt sequence ID | P10408 (amino acids 1-901) |
| Extinction coefficient (A_280_, 0.1% (w/v)) | 0.742 |
| M from chemical composition (Da) | 102022.99 (monomer) |
| Average C in merged data (mg/ml) | 0.5 |
| Solvent | 20mM HEPES, 100mM NaCl pH 8, 1mM TCEP |
|  |  |
| **SecAΔMBD** |  |
| Organism | *Escherichia coli* |
| Source | *Escherichia coli* |
| UniProt sequence ID | P10408 (amino acids 1-880) |
| Extinction coefficient (A_280_, 0.1% (w/v)) | 0.745 |
| M from chemical composition (Da) | 99665.28 (monomer) |
| Average C in merged data (mg/ml) | 0.5 |
| Solvent | 20mM HEPES, 100mM NaCl pH 8, 1mM TCEP |
|  |  |
| **SecAΔCTT** |  |
| Organism | *Escherichia coli* |
| Source | *Escherichia coli* |
| UniProt sequence ID | P10408 (amino acids 1-832) |
| Extinction coefficient (A_280_, 0.1% (w/v)) | 0.787 |
| M from chemical composition (Da) | 94329.44 (monomer) |
| Average C in merged data (mg/ml) | 0.5 |
| Solvent | 20mM HEPES, 100mM NaCl pH 8, 1mM TCEP |
| **SAXS Data collection parameters** |  |
| **Instrument** | ESRF BM29 BioSAXS |
| **Wavelength (Å)** | 0.919 |
| **Beamsize (µm)** | 700 x 700 |
| **Detector distance (m)** | 2.867 |
| **Q measurement range (Å^-1^)** | 0.0034-0.494 |
| **Absolute scaling method** | Comparison with scattering from 1mm pure H_2_O |
| **Monitoring for radiation damage** | Data frame-by-frame comparison |
| **Exposure time** | 1 s per frame |
| **Sample temperature (^o^C)** | 20 |
| **Software** | |
| **SAXS data reduction** |  |
| *I(q)* versus *q* | BsxCuBE |
| solvent subtraction, averaging and merging | PRIMUS (ATSAS 2.8.3) |
| **Extinction coefficient estimate** | ProtParam |
| **Basic analyses: Guinier, P(r), V_p_** | Primus (ATSAS 2.8.3), FoXS, ScÅtter |
| **Atomic structure modelling** | Gromacs, PyMOL 1.7 |
| **Three-dimensional graphic model representation** | PyMOL 1.7 |
| **SAXS Molecular weight** | SAXSMoW |
| **Structural parameters** |  |
| **SecA** |  |
| Guinier analysis |  |
| *I(0)* | 144.47 ± 0.076 |
| R_g_ (nm) | 4.22 ± 0.00 |
| q_min_ (nm^-1^) | 0.0174 |
| qR_g_ max (q_min_ = nm^-1^) | 1.29 |
| Coefficient of correlation, R^2^ | 0.99 |
| M from *(0)* (ratio to predicted)* | 224.7 (10.1 % to dimer) |
|  |  |
| **SecAΔMBD** |  |
| Guinier analysis |  |
| *I(0)* | 160.86 ± 0.045 |
| R_g_ (nm) | 4.15 ± 0.045 |
| q_min_ (nm^-1^) | 0.0129 |
| qR_g_ max (q_min_ = nm^-1^) | 1.29 |
| Coefficient of correlation, R^2^ | 0.92 |
| M from *(0)* (% discrepancy)* | 210.8 (5.73% to dimer) |
|  |  |
| **SecAΔCTT** |  |
| Guinier analysis |  |
| *I(0)* | 53.41 ± 0.086 |
| R_g_ (nm) | 4.51 ± 0.01 |
| q_min_ (nm^-1^) | 0.0304 |
| qR_g_ max (q_min_ = nm^-1^) | 1.28 |
| Coefficient of correlation, R^2^ | 0.95 |
| M from *(0)* (ratio to predicted)* | 232.3 (23.1 % to dimer) |
