# Supplementary material for: The C-terminal tail of the bacterial translocation ATPase SecA modulates its activity
Source: eLife. 2019 Jun 27;8:e48385. doi: 10.7554/eLife.48385 (PMC6620043; doi:10.7554/eLife.48385)
Supplement: Supplementary file 4. [file elife-48385-supp4.docx]

**Table describing fitting parameters of models of the *E. coli* SecA dimer.**

| **Model^a^** | **χ^2^** | ***c_1_^b^*** | ***c_2_*^c^** |
| --- | --- | --- | --- |
| **2FSG** | 3.66 | 0.99 | 3.36 |
| **2IBM** | 14.99 | 1.05 | 4.00* |
| **2IPC** | 24.08 | 1.05 | 0.51 |
| **1M6N** | 30.81 | 1.05 | 4.00* |
| **1NL3** | 25.01 | 1.05 | 1.73 |
| **6GOX** | 5.25 | 1.03 | 1.38 |

^a^SecA template structures used to generate models of the *E. coli* SecA homodimer

^b^Variable for scaling of the atomic radius, which also controls the excluded volume of the molecule (permitted range: 0.99 - 1.05)

^c^Variable used to adjust the difference between the densities of the hydration layer and the bulk water (permitted range: -2.0 - 4.0)

*Fitting reached limit of permitted range, which suggests overfitting of the data.
